# Marginal Cost of Carbon Sequestration Using Straw-Based Biochar in Great Britain

**DOI:** 10.1021/acs.est.5c11115

**Published:** 2026-01-09

**Authors:** Yuzhou Tang, Paul Wilson, Tim T Cockerill

**Affiliations:** 1 School of Mechanical Engineering, 120986 University of Leeds, Leeds LS2 9JT, U.K.; 2 School of Biosciences, 6123 University of Nottingham, Nottingham LE12 5RD, U.K.

**Keywords:** biochar production, marginal cost, spatial analysis, carbon sequestration, straw, scalability

## Abstract

Achieving the net-zero target of the United Kingdom requires substantial greenhouse gas removal (GGR) in addition to emission reductions. Biochar, a stable carbon-rich material produced through biomass pyrolysis, is an established GGR method. Straw is abundantly available in the UK and presents a viable option for large-scale biochar production. However, uncertainties regarding its feasibility remain, particularly concerning costs, spatial constraints, and facility construction. Here, we use a spatial model integrated with life cycle assessment and technoeconomic analysis to estimate the marginal cost curve for net carbon sequestration through straw-based biochar production in Great Britain (GB). Our findings reveal that straw-based biochar production in GB can achieve 0.6–1.9% of the UK’s 2050 carbon removal target at marginal costs below £75 per tCO_2_e. If higher-cost options are also included (i.e., without the £75 per tCO_2_e constraint), the total potential removal increases to 0.8–2.1%. The marginal costs are significantly influenced by the price and availability of feedstock, the value of byproducts, and the biochar yield. Our integrated spatial model helps identify optimal feedstock supply and production strategies, reducing costs and uncertainties in net carbon sequestration for biochar systems. This study elucidates the challenges and limitations of utilizing straw for large-scale biochar production in GB to support the climate change mitigation pathway.

## Graphical abstract

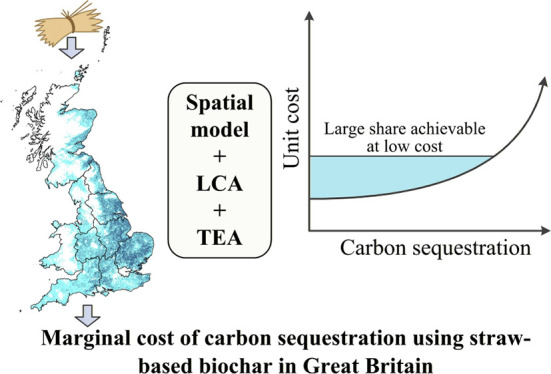

## 1 Introduction

Biochar has emerged as a promising greenhouse gas removal (GGR) technology, and assessments across regions indicate substantial annual removal potential.^1–6^ In the United Kingdom, national assessments suggest it could contribute up to 5 Mt CO_2_e per year toward the 2050 net-zero target.^6^ However, large-scale deployment remains limited by the absence of regionally resolved assessments of its economic feasibility.^6,7^ Existing studies often rely on simplified or indirect assumptions. On the one hand, studies focus on biochar derived from specific feedstocks and locations, without accounting for regional variation in feedstock availability.^8,9^ On the other hand, the few regional-scale assessments typically estimate theoretical biomass potential without considering the realistic availability of feedstocks or uncertainties in key economic drivers.^10,11^ These limitations result in an incomplete foundation for the strategic deployment of biochar in the UK, particularly with respect to feasibility, scalability, and cost-effectiveness.

Among various feedstocks, cereal straw offers particularly strong potential for biochar production in the UK, where approximately 3.2 million hectares of cereals are cultivated annually, yielding around 11 Mt of straw.^12,13^ Although straw is conventionally used for animal bedding, biofuel, and biomass energy, its storage and decomposition can lead to significantly greenhouse gas (GHG) emissions.^14^ An attractive alternative is to divert this residue into biochar production. Crucially, producing biochar from surplus straw does not require additional land in an attributional LCA perspective and avoids direct land-use conflicts, while also providing environmental cobenefits that enhance the value of agricultural residues, although diverting straw from existing uses may have indirect implications.^4,15^ In practice, soil application of biochar can reduce direct soil GHG emissions and nutrient losses, buffer pH, and improve soil structure and water-holding capacity, which in responsive soils translate into lower fertilizer demand and more stable yields.^15–17^ However, the biomass available for biochar is inherently limited and subject to competing demands from other GGR pathways or industrial uses.^6,18^ It is therefore essential to assess the environmental and economic viability of straw-based biochar systems within a broader portfolio of carbon removal options.

This paper aims to evaluate the potential of achieving carbon sequestration in Great Britain (GB) through biochar production from straw. By assessing its supply potential, regional distribution, and techno-economic viability, the study offers crucial insights into how straw-based biochar can contribute to GGR efforts, supporting the country’s transition toward net-zero emissions. The following research objectives are addressed: A spatial optimization approach is applied to quantify the marginal abatement cost and carbon removal potential of straw-based biochar production in GB. The analysis examines how variations in feedstock availability affect production efficiency and cost under different supply scenarios. Economic viability is further explored by analyzing the influence of reactive characteristics of the pyrolysis process (i.e., biochar yield, and the HHV of the bio-oil), external economic factors (i.e., transportation cost, capital cost, labor cost and field bean straw price) and the permanence coefficients for biochar carbon sequestration. A comparative assessment positions straw-based biochar alongside other GGR technologies to evaluate its policy relevance and competitiveness. Finally, the study identifies optimized strategies for feedstock allocation and processing site placement to support the scalable and cost-effective deployment of biochar as part of the UK’s net-zero strategy.

## 2 Materials and Methods

### 2.1 Research Framework

While slow pyrolysis technologies for biochar production are already well understood,^19–21^ their application remains largely limited to small-scale experimental or demonstration projects.^7^ Scaling up to a national-level biochar program will require a coordinated strategy for large-scale deployment. This requires addressing several key challenges in developing an effective biochar value chain: the feedstock characteristics (availability and distribution),^22^ the characteristics of processing sites (number, location, and scale),^23^ the distance between feedstock sources and processing sites,^24^ the achievable net carbon sequestration,^10^ and the associated costs.^25^

This study addresses these challenges by analyzing the marginal cost of net carbon sequestration from large-scale straw-based biochar production across GB (Figure 1). The analysis begins with the estimation of straw yield for five major arable crops based on cultivation conditions. A significant portion of straw is retained on farms for soil-health maintenance and for bedding and feed.^26^ Availability for biochar is therefore represented by two scenarios derived from end-use statistics:^27^ a low supply scenario utilizing 22% of straw primarily from nonagricultural uses and a high supply scenario utilizing 57% by incorporating all straw sold off farm. A uniform utilization rate is applied across GB, acknowledging that region-specific variation may exist but cannot be parametrized with current data.

**Figure 1:**
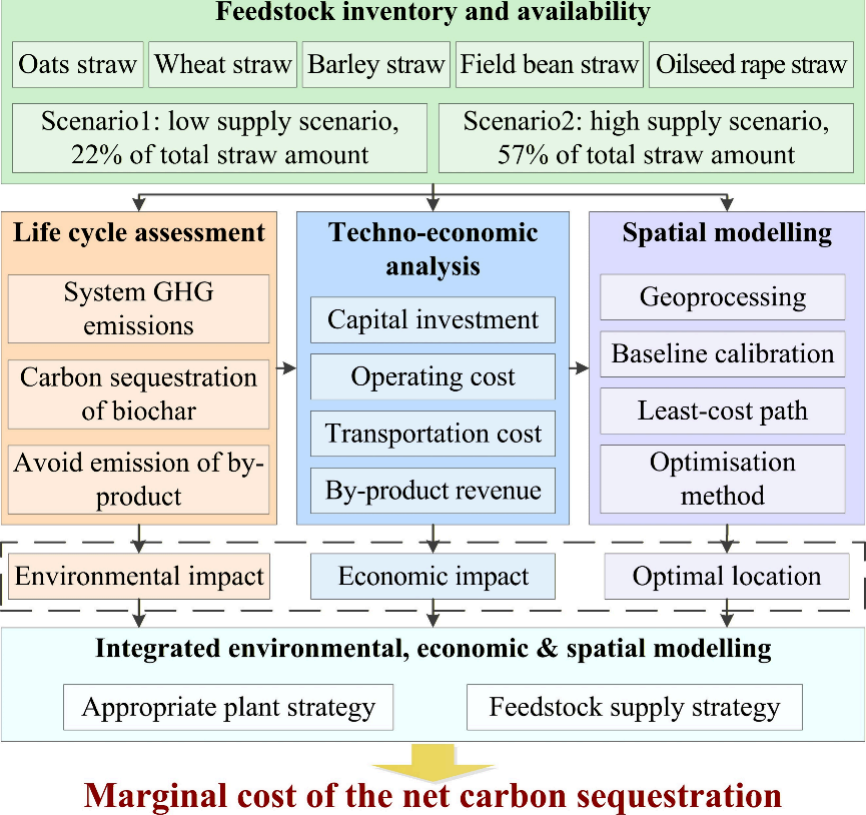
Technical path for marginal cost calculation.

We then assess the marginal cost of net carbon sequestration of large-scale biochar production from straw for the two scenarios in GB. The system boundary is cradle-to-gate, including straw supply, transportation of the feedstock to processing sites, and biochar production, with downstream system expansion incorporating the climate credit of biochar and fossil-fuel displacement of bio-oil. Other downstream processes, including application logistics, soil GHG fluxes and agronomic responses, are excluded from this boundary, which represents a limitation in capturing the full range of impacts associated with biochar application. The functional unit is one year of operation in the research area. Building on this, the study evaluates the GHG emissions and carbon sequestration potential of the biochar system, including the carbon offset benefits of byproducts like bio-oil that may displace fossil fuels. Economic performance is then assessed by examining feedstock supply costs, transportation expenses, capital investment, operation expenditure, and potential revenues from byproducts.

Due to the spatially dispersed nature and large areal extent of straw supply, and because early biochar markets are expected to be highly local owing to transport costs,^28^ each region is modeled separately. The regions include East of England (EE), East Midlands (EM), North East (NE), North West (NW), Yorkshire (YO), West Midlands (WM), South East (SE), South West (SW), Scotland (SC) and Wales (WA).

A spatial optimization model is applied to identify the most cost-effective locations for processing sites and to determine feedstock clustering patterns that minimize mass-distance under different deployment scales. By integrating feedstock dynamics, facility siting, environmental outcomes, and economic costs, this framework provides a comprehensive assessment of the technical and economic feasibility of scaling up straw-based biochar production in GB. By explicitly accounting for geographic constraints, the analysis enables the development of locally appropriate configurations,^29^ thereby supporting regionally tailored implementation strategies and informing the UK’s broader GGR efforts.

### 2.2 Data for Straw Yield in Great Britain

Based on the actual farming conditions, we select the straw from five major crops for evaluation as biochar feedstock: wheat, barley, field bean, oats, and oilseed rape. Feedstock availability is represented by two scenarios: low supply scenario (22%) and high supply scenario (57%). Accordingly, the scenario-defined proportion of straw is taken as utilized for biochar production. Utilizing the spatial model, crop data,^30^ and crop straw production per hectare (Table 1), we can determine the total harvestable straw quantity for each region and the proportion of each crop type.

**Table 1:** Straw Yield of Each Crop per Hectare

crop type	straw yield (t/hectare)	reference
spring barley	2.8	^31^
winter barley	2.7	^31^
wheat	4	^31^
oats	1.6	^31^
oilseed rape	1.5	^32^
field beans	2.39	^33^

### 2.3 Life Cycle Assessment

Life Cycle Assessment (LCA) of GHG is widely used to quantify climate impact and propose emission reduction strategies.^34^ The study applies attributional LCA to evaluate the climate impact for each processing site. This method is extensively employed in evaluating the challenge of net carbon sequestration for biochar production.^35–37^ In this study, net carbon sequestration is defined as the long-term biochar storage credit minus cradle-to-gate burdens. Avoided emissions from bio-oil use are excluded from the LCA because the analysis focuses solely on carbon removal, and such substitution credits depend on the specific utilization pathway and may diminish in relevance as energy systems decarbonise.

The life cycle inventory of the biochar production includes three main stages: feedstock supply, transportation, and biochar production. Data for feedstock supply and transportation are derived from the spatial model results. The Ecoinvent Database in SimaPro (2022) is used to assess GHG emissions per unit of feedstock collected and per unit of mass distance traveled. The calculation of the life cycle GHG emissions (*GHG*_*tot*_), is given by

$${GHG}_{tot}={GHG}_{supply}+{GHG}_{trans}+{GHG}_{prod}$$ 1

where *GHG*_*supply*_ represents supply emissions, *GHG*_*trans*_ represents transportation emissions, and *GHG*_*prod*_ represents production emissions.

The consumption of utilities (electricity, natural gas, and water) for the biochar production process is based on data from a 5-t-per-hour unit provided by Beston Ltd.^38^ and scaled according to size factors.^39^ As the produced biochar is intended for carbon sequestration, a pyrolysis temperature of 500 °C is assumed to meet stability requirements. The moisture content, pyrolysis products, and characteristics of the biochar produced from various crop straw types under 500 °C are shown in Table 2. GHG emissions from electricity consumption are calculated using the 2023 average emission factor for UK electricity.^40^ Natural gas is assumed to be pure methane and fully combusted, with the resulting GHG emissions calculated as 11/4 of the mass of the natural gas. The emissions from the straw pyrolysis are not included in the lifecycle GHG accounting, as they are considered biogenic carbon emissions.^41,42^

**Table 2:** Pyrolysis Characteristics of Crop Straw

	barley straw^48^	wheat straw^48^	oat straw^48^	oilseed straw^49–51^	field bean straw^52^
moisture (%)	2.7	5.3	4.4	2.9	4.3
biochar yield (%)	33.5	36.2	32.7	32.7	30.0
liquid yield (%)	35.8	40.5	40.0	49.5	44.9
syngas yield (%)	30.7	23.3	27.3	17.9	25.1
biochar carbon content (%, dry basis)	72.6	67.9	66.4	67.3	72.1
H:C molar ratio	0.51	0.52	0.42	0.46	0.43

In this study, we assume that all biochar produced is applied to soil for carbon sequestration. In the UK, biochar application to agricultural soils is subject to a regulatory cap of 1 t per hectare per year.^43^ Under the high-supply scenario, applying straw-derived biochar within its sourcing areas implies about 0.57 t per hectare per year, which is below this cap, so application limits are not binding at the national scale in our analysis. The carbon sequestration potential is assessed using the stable carbon content remaining in biochar after 100 years, following the 100-year Global Warming Potential (GWP100) approach. The study selects the H:C molar ratio as the criterion for assessing biochar stability. 60% of the carbon within the biochar is considered stable after 100 years when the H:C molar ratio is less than 0.6.^44^ The carbon sequestration amount (*CS*) is calculated as

$$CS=\sum biochar_{i}*C_{i}*p_{i}$$ 2

where i represents the straw type, *biochar*_*i*_ represents the mass of biochar produced from straw type i, *C*_*i*_ represents the carbon content of the biochar produced from straw type *i*, and *p*_*i*_ is the long-term stable carbon fraction. In the baseline assessment, *p*_*i*_ is set to 0.60 for all biochars with H:C < 0.6.

For context, H:C based formulations reported by Woolf et al. (2021) typically imply permanence greater than 0.70 over 100 years for the feedstock types used in this study in temperate soils.^45^ In a sensitivity analysis, we therefore recalculate *p*_*i*_ using the their formulation to examine how adopting the newer permanence estimates would affect the unit cost of the net carbon sequestration.

In the GB context, we assume stand-alone pyrolysis deployment because colocation with biogas plants is neither required by current regulations nor commonly observed in existing commercial practice. Consequently, full valorization of syngas heat is not included in the base case. This likely understates the byproduct benefit. If the remaining syngas could be exported, the total cost would be further reduced. Bio-oil, however, is considered a substitute for fossil fuels, and the fuel replacement (*FR*) benefits are calculated according to the method outlined by Brassard.^46^ The calculation is given by

$$FR=\sum liquid_{i}*\frac{HHV_{liquid}}{HHV_{fueloil}}*emission_{fueloil}$$ 3

where *liquid*_*i*_ represents the mass of bio-oil produced from straw type i. *HHV*_*liquid*_ is the higher heating value (HHV) of the liquid product, which is 12.7 MJ/kg.^46^ *HHV*_*fueloil*_ is the HHV of #2 fuel oil, which is 36.6 MJ/L,^47^ and *emission*_*fueloil*_ is the GHG emission reduction per unit of #2 fuel oil replaced, which is 2.57 kg CO_2_e/L.^46^

### 2.4 Technoeconomic Analysis

Techno-economic analysis (TEA) is a crucial step toward scaling up the technology to an industrial level by evaluating the economic performance of the process, system configuration and energy usage.^53^ This method can address the cost challenge associated with biochar production.^54–56^ Although these methods have been applied in biochar research, the extant literature predominantly reports average biochar cost or single-site case studies. To our knowledge, only one study has produced spatially explicit, site-resolved marginal-cost estimates by ordering candidate processing sites according to biochar cost derived from local feedstock attributes and transport/siting constraints.^57^ TEA is employed to estimate the investment required for deployment, incorporating production costs, byproduct revenues and process performance parameters such as the biochar yield and the HHV of the bio-oil. It helps identify key cost drivers and sensitivities for large-scale biochar production.

Economic parameters for capital expenditure (CAPEX), fixed operating expenditure (fixed OPEX) and variable operating expenditure (variable OPEX) follow previous work.^57^ CAPEX includes funds required to purchase land, design and purchase equipment, structures, and buildings as well as to bring the facility into operation.^58^ Fixed OPEX includes the expenses of operating labor, maintenance and depreciation, as well as general overhead expenses and plant indirect expenses. Variable OPEX comprises process utilities (electricity and water) during production. Revenue from coproducts is included in the analysis, with bio-oil income valued at £10/GJ.^59^ It is assumed that the plant would have a lifespan of 8 years and operate 7,200 h annually.

The cost of feedstock supply (*Cost*_*supply*_), is calculated by

$$\cos t_{supply}=\sum feedstock_{i}*price_{i}$$ 4

where *feedstock*_*i*_ represents the mass of straw type *i* consumed by the processing site, as derived from the spatial model simulation. The unit price of each straw type (*price*_*i*_), is based on the Hay and Straw Prices statistics,^60^ using the average prices from April 2019 to March 2024. The maximum and minimum prices over these five years were used to assess the impact of straw price variability on the results. Data are available for barley, wheat, and oilseed rape straw. It was assumed that oats straw follows the same price trend as barley, while the price of field bean straw was based on Pea and Bean Straw Price Guidance.^61^ Given that field bean straw constitutes only 5% of the total raw material and lack of a time series, a constant price is adopted. To avoid implicitly assuming zero volatility, field-bean price variability is examined through a sensitivity analysis. The prices of feedstock are shown in Table 3.

**Table 3:** Straw Prices Considered in the Study

straw type	price (ave) £/t	price (min) £/t	price (max) £/t
barley straw	64.5	44.0	107.0
wheat straw	56.7	38.0	104.0
oats straw	64.5	44.0	107.0
oilseed rape straw	59.0	44.0	81.0
field bean straw	65.0		

The cost of the transportation stage (*Cost*_*trans*_), is estimated using the mass distance results (*trans*) from the spatial model and a unit transportation cost of £0.22/t.km derived from the UK DAC and GGR Programme (Phase 1) biochar projects,^62^ as given by

$$\cos t_{trans}=trans*0.22$$ 5

We calculate the life cycle costs and byproduct revenues to assess the economically feasible price of biochar and the marginal cost to achieve net carbon sequestration. In this study, the marginal cost denotes the unit cost (£ per t CO_2_e) of the additional net removal delivered by the next processing site in a cost-ordered deployment. Because sites vary in unit cost and removal capacity due to regional differences in feedstock availability, transport distance and plant scale, the marginal cost tends to rise as lower-cost sites are utilized. In addition to examining the impact of straw price and supply scenarios, a sensitivity analysis is conducted to evaluate the influence of various factors on the biochar price and the marginal cost of net carbon sequestration. This analysis considers seven key variables: biochar yield, byproduct HHV, unit transportation cost, CAPEX, labor cost, field bean straw price, and permanence coefficient. Each selected variable represents a key cost component, ensuring that the analysis reflects the main sources of economic uncertainty in the straw-to-biochar pathway.

### 2.5 Spatial Analysis Model

Spatial modeling, based on geographic information systems (GIS), is a method for understanding and analyzing economic and environmental phenomena using data or information related to specific locations. This approach has been applied to various engineered GGR technologies^57,63–66^ and has proven effective in addressing challenges related to feedstock,^64,67^ processing sites,^66,68^ and transportation distance.^29,57^ All GIS work is conducted using QGIS 3.38 software, following a similar spatial modeling approach as described in the previous work.^57^ Specifically, we first construct a 1km*1km feedstock layer based on the crop map.^26^ This layer is modified and validated using OS OpenMap - Local,^69^ Rural Urban Classification^70^ and Google satellite map.^71^ The annual yield of each straw type is calculated for each grid cell. Next, we create a 5km*5km plant location layer. Then, using the roadmap^72^ and the ″shortestpathpointtolayer″ function in the PyQGIS toolbox, we determine the shortest transport distance matrix between each grid cell of feedstock layer and each grid cell of plant location layer. Finally, particle swarm optimization (PSO), a widely used nonlinear optimization method,^73^ is employed to select the optimal processing site locations to minimize transport distance. We define the optimization objective as minimizing the total mass-distance. Using the shortest transport distance matrix as constraints, PSO with randomly initialized candidate site coordinates is applied to determine the optimal locations for processing sites, assuming between 1 and 10 plants.

Through spatial modeling, we obtain the results for different numbers of processing sites, including the scale of each processing site, the types of straw processed, and the feedstock supply clusters.

## 3 Results

### 3.1 Spatial Distributions of Feedstock Supply in Great Britain

One challenge in scaling up biochar production using straw is spatially matching feedstock supply with processing site locations. Figure 2 shows the spatial distribution of straw feedstocks from farms across Great Britain. The spatial distribution indicates that GB is expected to harvest 11 Mt of straw annually, with wheat straw and barley straw accounting for 86% of the total amount, primarily distributed in the eastern and southern regions. Availability for biochar is evaluated under the two scenarios defined in the Methods: a low supply scenario (22% of total straw) and a high supply scenario (57%).

**Figure 2:**
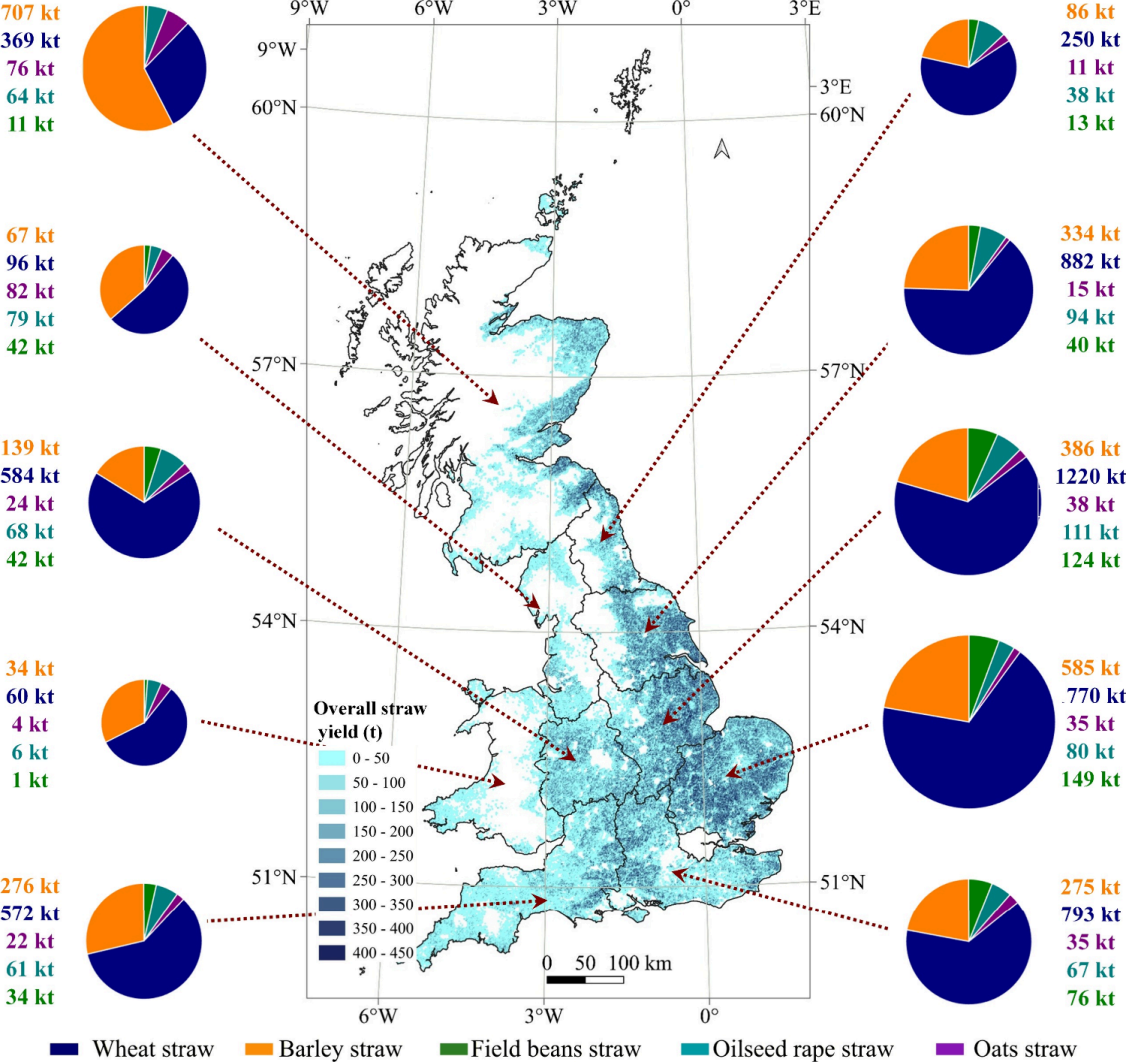
Spatial distribution of straw feedstocks from farms across GB, showing the overall annual straw yield (t) by region. The background map illustrates the total yield density, with darker shades indicating higher straw yields. The pie charts represent the composition of straw types in each region, including wheat straw (blue), barley straw (orange), field beans straw (green), oilseed rape straw (yellow), and oats straw (purple). The size of each pie chart is proportional to the total straw yield of the respective region, providing a detailed overview of straw distribution across Great Britain.

Using spatial optimization, feedstock supply is assigned to processing sites in a cost-minimizing manner, with the resulting configuration presented in Figure 3. The resulting feedstock supply clusters are illustrated by the corresponding colors surrounding each processing site, denoted by stars. The key results of biochar production under the two scenarios are presented in Table 4 for clarity and comparison. We estimate that producing biochar in GB using straw for nonagricultural purposes (low supply scenario) would require a total of 23 processing sites, handling 2.5 Mt p.a. of feedstock, yielding 0.8 Mt p.a. of biochar, at a biochar cost of £100.8/t. If the feedstock supply is expanded to include all sold straw (high supply scenario), the number of processing sites increases to 39, with the feedstock quantity rising to 6.2 Mt pa, biochar yield increasing to 2.0 Mt pa, and the biochar cost decreasing to £88.0/t.

**Table 4:** Summary of Biochar Production Results Under Low and High Supply Scenarios

scenario	number of processing sites	feedstock quantity (Mt p.a.)^a^	biochar yield (Mt p.a.)	Biochar cost (£/t)
low supply scenario	23	2.5	0.8	100.8
high supply scenario	39	6.2	2.0	88.0

a *Note*: ″Mt p.a.″ refers to million t per annum.

**Figure 3:**
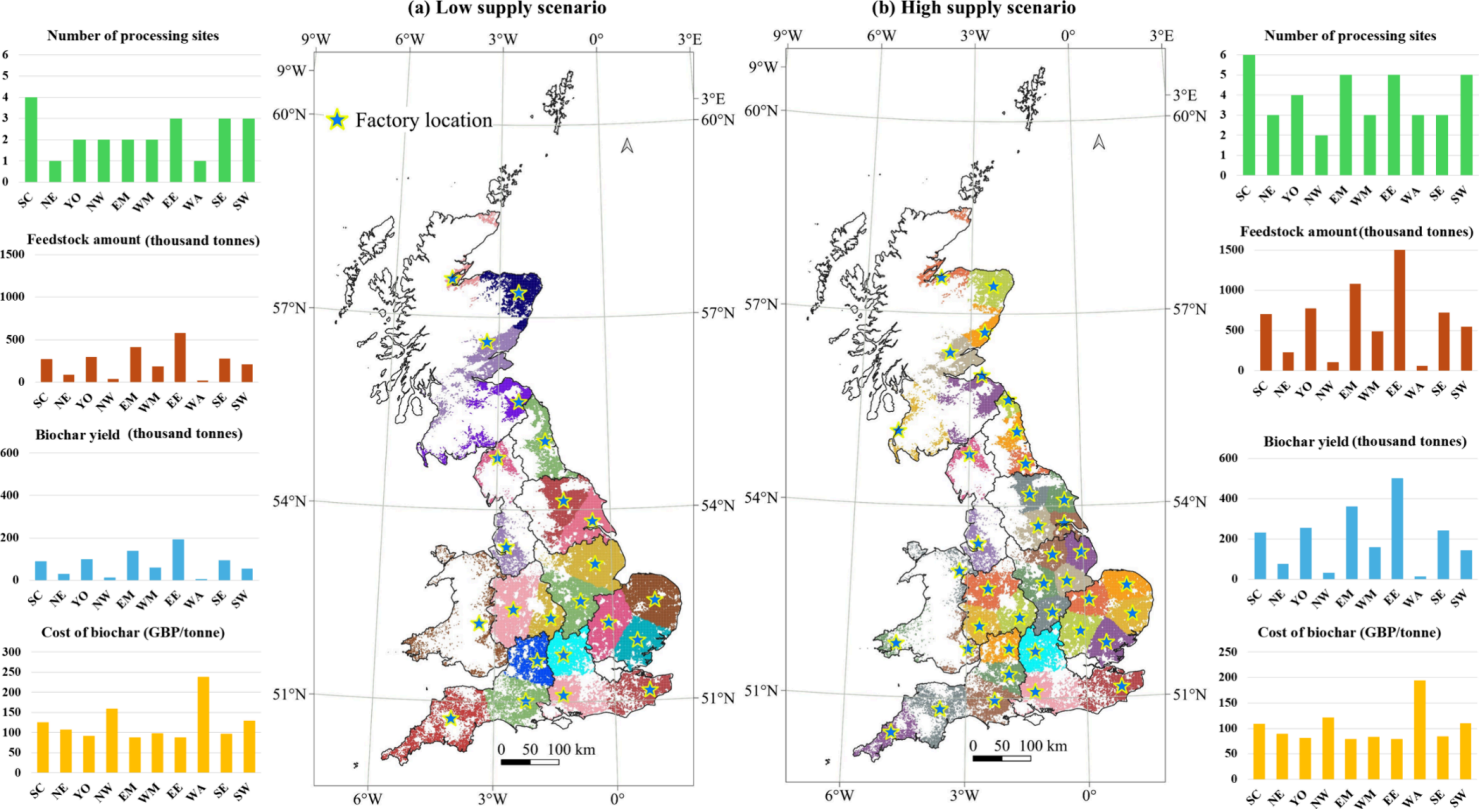
Spatial results of the plant strategies and feedstock supply strategies in GB. The star symbols in the two central diagrams represent the locations and number of factories in each region, corresponding to the plant strategy. The colored areas surrounding each processing site indicate the clustering of feedstock supply, illustrating the feedstock strategy. (a) low supply scenario, (b) high supply scenario.

With respect to the chosen location and numbers of processing sites, the primary factors are the availability and spatial distribution characteristics of the feedstock in each region. Comparing the two scenarios, as the proportion of feedstock availability increases, the rise in transportation costs outweighs the cost reductions associated with larger plant scales (such as improved energy efficiency and lower unit labor costs). As a result, more processing sites are required. Since straw distribution in GB is predominantly concentrated in the eastern and southern regions, most processing sites in both scenarios are located in these regions, where the density of feedstock distribution is higher.

Biochar production is chiefly determined by the feedstock availability in each region. The four eastern regions (SC, SE, EE, and EM) hold 78.9% of the total feedstock, allowing them to produce the highest amounts of biochar. Conversely, the western regions of WA, NW, and NE have the least feedstock distribution and thus generate the smallest amounts of biochar. The regional biochar cost is also closely related to the spatial distribution and density of the feedstock. For instance, although SC has relatively high feedstock availability, its wide distribution leads to higher transportation costs, resulting in a higher biochar cost. In contrast, the five regions with higher feedstock density (SE, EE, EM, YO, and WM) benefit from lower biochar cost mainly due to shorter transportation distances. In these areas, biochar is produced at costs below £98/t in low supply scenario and below £85/t in high supply scenario, accounting for 75% of the total biochar produced.

### 3.2 Marginal Cost of Net Carbon Sequestration

Figure 4 presents the marginal cost results for net carbon sequestration. The following subsections summarize patterns by sequestration level, feedstock supply, regional variation and price sensitivity.

**Figure 4:**
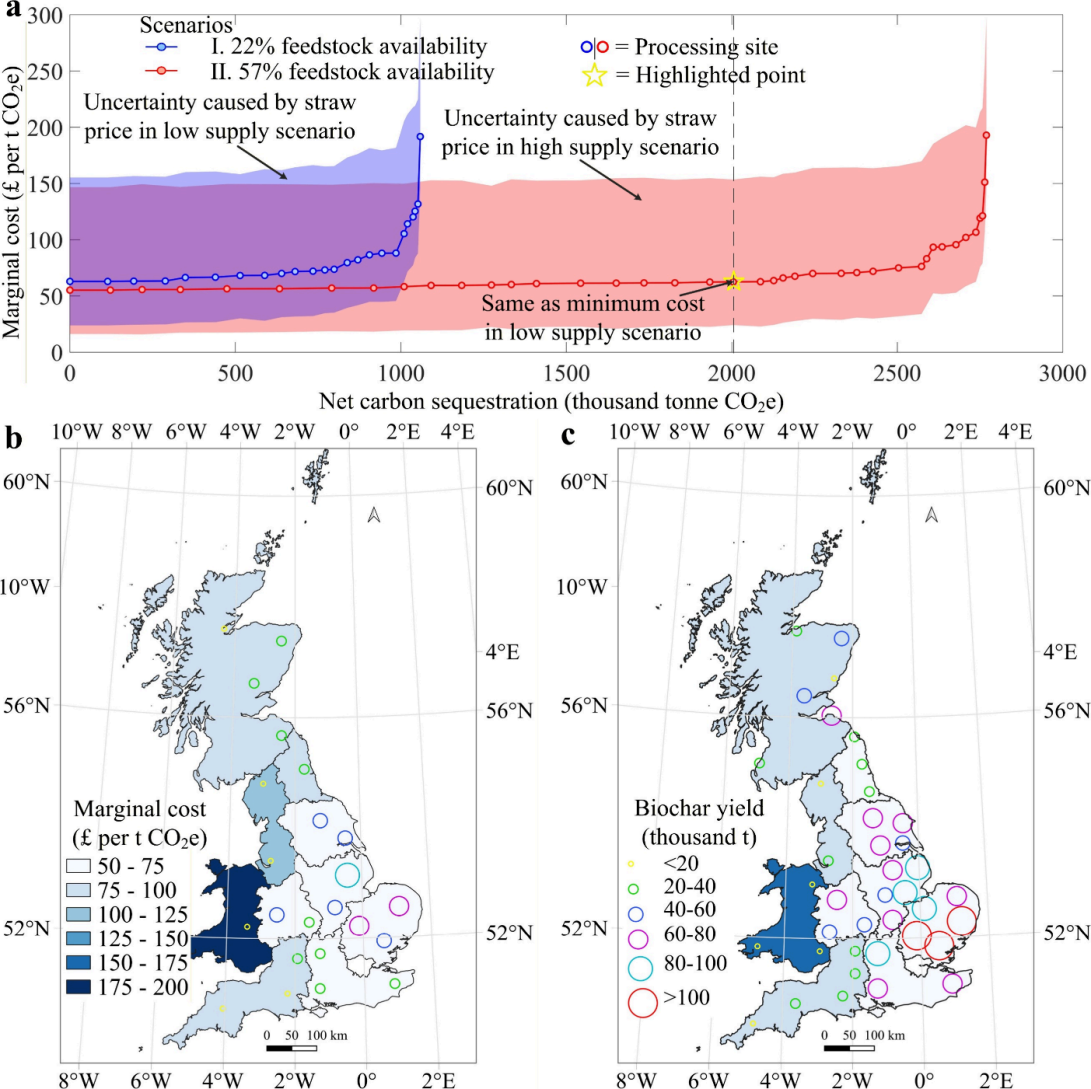
Marginal cost curves of biochar production in GB. (a) Marginal cost curve illustrating the relationship between cumulative net carbon sequestration and marginal abatement costs under both scenarios. (b) Spatial distribution of processing sites in the low supply scenario. (c) Spatial distribution of processing sites in the high supply scenario. The legends in panels b and c are applicable to both panels.

#### 3.2.1 Diminishing Returns at Higher Sequestration Levels

In the low supply scenario, shown by the blue dotted line in Figure 4a, the marginal cost rises from £63 to £75 per t CO_2_e as the sequestration level increases from zero to 0.80 Mt CO_2_e per year. Further increases in sequestration to 1.01 and 1.06 Mt CO_2_e per year raise the marginal cost to £105 and £192 per t CO_2_e, respectively. This demonstrates a significant rise in marginal cost beyond 0.80 Mt CO_2_e pa, with diminishing returns, especially for the last 0.05 Mt CO_2_e pa, which requires an additional £87 in marginal cost. In the high supply scenario, shown by the red dotted line in Figure 4a, the marginal cost increases from £55 to £64 per t CO_2_e as the sequestration level rises to 2.13 Mt CO_2_e per year. Raising the level further to 2.77 Mt CO_2_e per year increases the marginal cost to £193 per t CO_2_e. This likewise indicates a pronounced rise beyond 2.13 Mt CO_2_e pa.

#### 3.2.2 Impact of Feedstock Supply

Unsurprisingly, the results show that increased feedstock availability reduces marginal costs. The high supply scenario assumes 57% of straw is used for biochar production. Here, to achieve a net carbon sequestration from 0 to 2.08 Mt CO_2_e pa, the marginal abatement cost increases from £55 to £63/t CO_2_e, which matches the lowest marginal cost in low supply scenario. Further increasing the sequestration level to 2.50, 2.76, and 2.77 Mt CO_2_e p.a. raises the marginal cost to £75, £107, and £193/t CO_2_e, respectively.

#### 3.2.3 Regional Variation in Marginal Costs

Regional marginal costs reflect two modeled drivers: economies of scale and logistics. Areas with higher feedstock density support larger plant capacities, lowering unit processing costs through the CAPEX scaling factor, as observed in EM, EE and YO. In contrast, WA, characterized by lower feedstock density, entails longer average haul distances and smaller feasible plant sizes, which raise both unit transport and processing costs. These mechanisms operate in both the low and high supply scenarios. Higher availability primarily enables larger installed capacities and shorter average hauls, thereby shifting costs downward while preserving the same spatial logic.

#### 3.2.4 Influence of Feedstock Price Fluctuations

Straw prices in GB are relatively volatile owing to year-on-year fluctuations in demand and production, in many cases weather related. It is important therefore to explore the impact such fluctuations might have on marginal costs. Using the highest and lowest straw costs from the last five years shows that feedstock price fluctuations have a substantial impact on marginal cost. Uncertainty is reported as the lower and upper deviations from the baseline (mean-price) estimate. In the low supply scenario, the deviation pair narrows from – 62%/+147% to – 24%/+55% with increasing net sequestration levels. In the high supply scenario, it narrows from – 71%/+166% to – 24%/+54%. This reduction in uncertainty is primarily due to the fact that achieving higher sequestration levels requires additional investments in new processing sites, increased transportation capacity, and higher operational costs. These fixed and variable expenses become more significant as the scale of production expands, thereby diminishing the relative influence of feedstock price fluctuations on the overall cost. At the minimum feedstock price, the cost of using all available straw for biochar production in GB falls within the indicative range of £14–130 per t CO_2_e reported in the *Greenhouse Gas Removal (GGR) policy options*.^74^ This range synthesizes UK-focused estimates derived under varying assumptions across the production to application chain and should be interpreted as an order-of-magnitude guide rather than a like-for-like benchmark. However, at the maximum feedstock price, the overall costs consistently exceed this range, highlighting the sensitivity of the system to feedstock price variability.

### 3.3 Emissions, Cost and Sensitivity of Biochar Production

We assessed the emissions and costs associated with biochar production across different regions in GB. This evaluation encompassed the entire production lifecycle, including feedstock supply, transportation, and processing stages, to quantify GHG emissions and costs.

The LCA results reveal the key components of emissions, the carbon sequestration by biochar, and the fuel replacement benefits from bio-oil (Figure 5). Overall, GHG emissions from the lifecycle process are negligible compared to the carbon sequestration and fuel replacement benefits. System emissions are dominated by feedstock supply, followed by transport and production. Scenario-specific shares are shown in Figure 5(a). When comparing the two scenarios, as feedstock availability increases, the share of emissions from straw supply rises, while emissions from transportation and production decrease due to shorter transportation distances and greater production efficiency.

**Figure 5:**
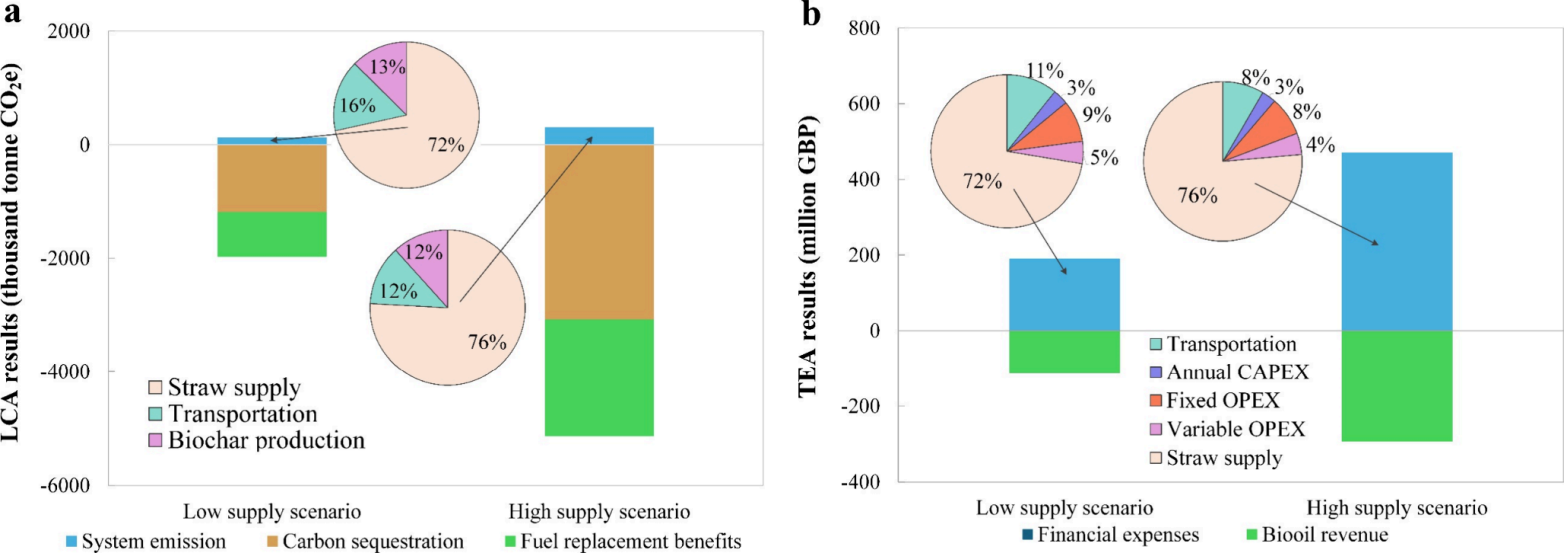
Life cycle GHG emissions (a) and costs (b) results of biochar production.

The TEA results illustrate the costs associated with biochar production and the potential revenue from byproducts (Figure 6). However, revenue from byproducts alone does not cover production costs, highlighting the need for extra income. In both scenarios, straw supply constitutes the highest cost, exceeding 70%, followed by transportation, fixed OPEX, variable OPEX, and CAPEX. The scenarios reveal that with greater feedstock availability, plants operate at larger capacities and higher utilization, reducing unit CAPEX and OPEX via economies of scale and improving average logistics intensity per tonne. Since the feedstock purchase cost per tonne is held exogenous and scales with throughput, its proportion of the biochar cost increases even as total biochar cost declines. The byproduct bio-oil can generate substantial economic returns, which can cover 60% of the total financial expenses.

**Figure 6:**
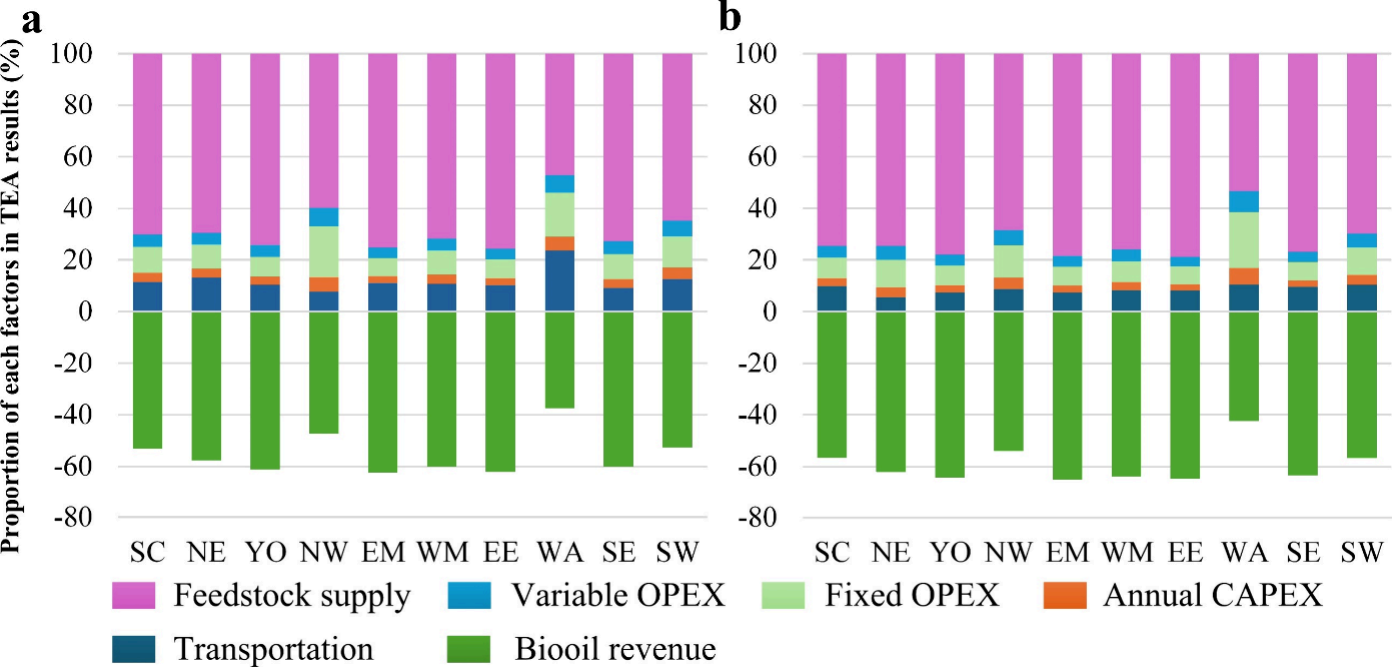
Techno-economic cost of biochar production under low (a) and high (b) supply scenarios.

This analysis compares changes in unit net carbon sequestration cost across the supply scenarios when each parameter is varied individually, holding all other inputs constant. For all parameters except the permanence coefficient, a 20% reduction is applied to assess sensitivity. The permanence coefficient is evaluated separately using the updated formulation from Woolf et al. (2021).^45^ The findings indicate that reducing reactive characteristics results in a significant increase in biochar cost (approximately 30%), while reducing external factors causes a moderate decrease (around 5%). In addition, replacing the baseline permanence coefficient with the higher permanence estimate derived from Woolf et al. (2021) increases the credited sequestration and correspondingly reduces the unit net carbon sequestration cost by 27%. Therefore, improving biochar yield and the quality of bio-oil, while maintaining biochar stability, is crucial for the large-scale production and cost reduction of biochar. The influence of the permanence coefficient on the results is particularly notable, and the use of a conservative baseline assumption means that the economic performance reported here is likely understated. Adopting a higher stability estimate would further improve the overall economic outcomes. Under large-scale biochar deployment, economies of scale reduce the unit contributions and sensitivities of CAPEX and labor cost to total cost, while the spatial analysis model further diminishes logistics impacts.

Increasing feedstock supply reshapes the cost structure and, in turn, the sensitivity of biochar cost to individual drivers. This can be seen by comparing Figure 7a and Figure 7b, where the change in color tones for the same region and variable reflects the shift in sensitivity under higher feedstock supply. As more processing sites become viable, average haul distances per tonne fall, the transport cost share declines, and the biochar cost becomes less sensitive to a 20% change in transport cost. Greater availability also permits larger capacities and higher utilization, which lowers the unit contributions of CAPEX and labor through economies of scale; correspondingly, sensitivities to CAPEX and labor change little or decline. At the same time, higher throughput increases bio-oil output and revenue, raising the cost’s responsiveness to bio-oil HHV, while the relative influence of biochar yield diminishes as other components are diluted.

**Figure 7:**
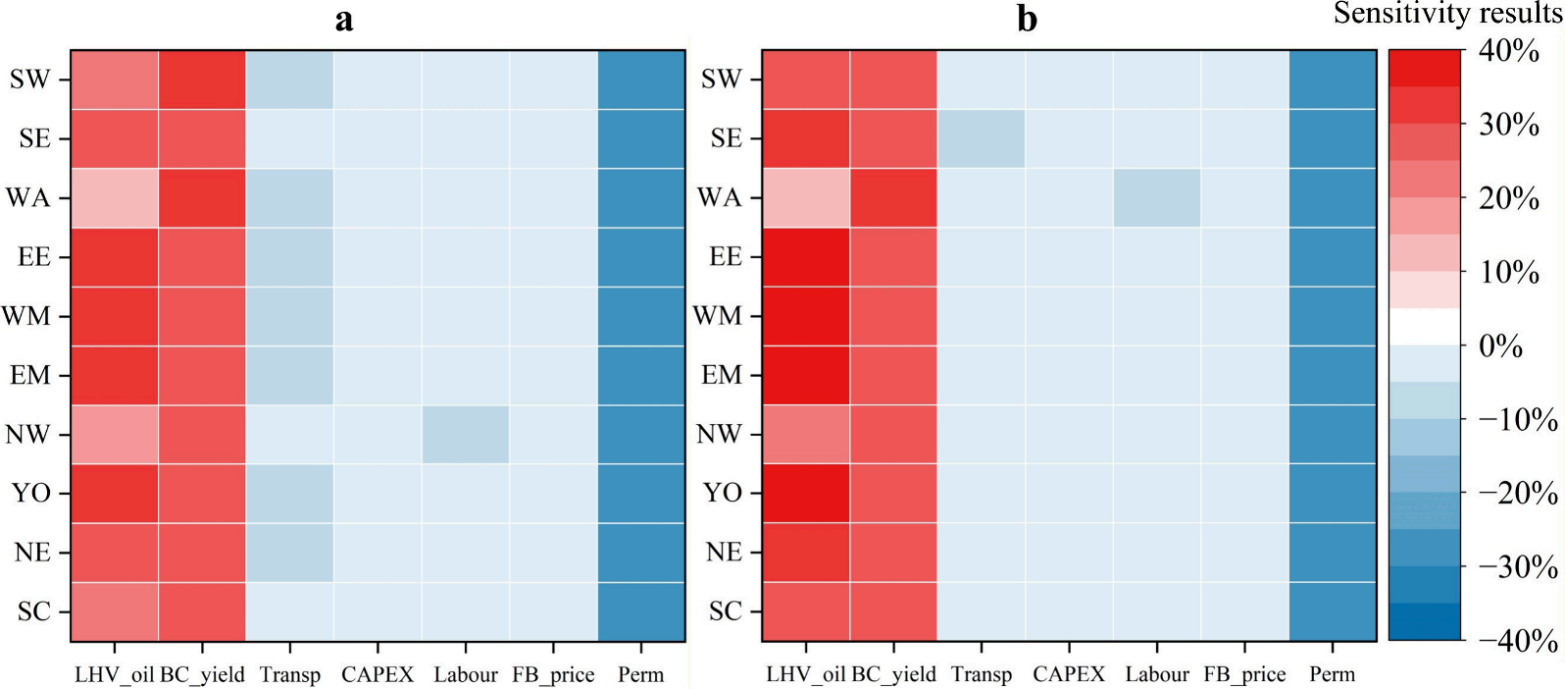
Impact of reactive characteristics and external factors on unit net carbon sequestration cost, (a) low supply scenario; (b) high supply scenario. Abbreviations: LHV_oil: lower heating value of bio-oil; BC_yield: biochar yield; Transp: transport cost; CAPEX: capital expenditure; Labour: labor cost; FB_price: field bean straw price; Perm: permanence coefficient.

### 3.4 Role of Biochar in Net Zero

Based on the marginal cost curves in Figure 4, we estimated the potential contribution of straw-based biochar production toward the net zero target. In low supply scenario, an annual investment of £54 million in biochar production can achieve approximately 0.8 Mt CO_2_e p.a. of net carbon sequestration, contributing 0.6% toward the UK’s goal of removing 130 Mt CO_2_ annually by 2050. Increasing the investment to £78 million can raise this contribution to 0.8%. If more feedstock is available for biochar production (high supply scenario), an annual investment of £152 million can achieve 2.5 Mt CO_2_e p.a. of net carbon sequestration, representing 1.9% of the total target. Further increasing the investment to £178 million can raise this contribution to 2.1%. Investment figures represent the supply side costs when bio-oil revenue is included as a coproduct credit. Economic viability therefore depends on how this net cost compares with the societal benefit of carbon removal, which is represented by the applicable carbon value. Private investment becomes attractive when the prevailing carbon value meets or exceeds the marginal cost after accounting for bio-oil revenue. If the carbon value is lower, additional policy support is required.

Additionally, the byproducts of biochar production can significantly contribute to avoided emissions by serving as a fuel substitute. In low supply scenario, bio-oil can replace up to 11.3 million GJ of energy, approximately 0.6% of the 2050 sustainable energy target for power generation, equivalent to about 0.8 Mt CO_2_e p.a. of avoided emissions. In high supply scenario, the bio-oil can replace 29.3 million GJ of energy, meeting 1.5% of the sustainable energy target.

Our analysis indicates that the estimated cost of carbon sequestration through biochar ranges from £55 to £193 per t CO_2_e. This range reflects heterogeneity in local feedstock availability, spatial distribution and feedstock prices. The level of government support for carbon sequestration should be assessed against the applicable carbon value, which is around £78 per tCO_2_e in 2030 according to the UK Government’s traded carbon values,^74^ and this condition is met when the site-specific marginal cost does not exceed that value. Table 5 illustrates the indicative costs of various GGR technologies.^75^ Despite considerable uncertainties, biochar demonstrates significant potential competitiveness compared to bioenergy with carbon capture and storage (BECCS), enhanced weathering, and direct air carbon capture and storage (DACCS), all of which are GGR methods yet to be demonstrated at scale. In comparison to nature-based approaches such as soil carbon sequestration and afforestation, biochar has a higher unit carbon sequestration cost, although it provides significantly longer carbon storage lifetimes than these options. However, about 80% of the carbon sequestration in both scenarios can be achieved at around cost of £75 per t CO_2_e, which is comparable to the cost of habitat restoration. Our findings have similar ranking to Smith’s.^76^ The results suggest that straw has measurable net carbon sequestration potential, positioning biochar as an economically viable option among engineered GGRs.

**Table 5:** Abatement Cost Comparison of GGR Technologies, Ranked by Min Cost (Lowest to Highest)

GGR technology	min cost (£/t CO_2_e)	max cost (£/t CO_2_e)	source
afforestation	2	23	^75^
soil carbon sequestration	4	20	
habitat restoration	8	78	
enhanced weathering	39	390	
biochar high supply scenario (80% yield)	55	68	this work
biochar high supply scenario (100% yield)	55	193	
biochar low supply scenario (80% yield)	63	80	
biochar low supply scenario (100% yield)	63	192	
BECCS	80	230	^75^
DACCS	160	470	

### 3.5 Policy Implication

To fulfill the commitments of the Paris Agreement, it is essential to assess and develop scalable, viable GGR methods. The UK Government’s *Clean Growth Strategy* and the Royal Society and Royal Academy of Engineering have both emphasized the importance of formulating GGR strategies to achieve net-zero emissions targets. Biochar, a well-established GGR method, can stably sequester carbon from biomass while producing low-carbon energy. Our spatial optimization results indicate that utilizing domestic straw for biochar production could realistically achieve 21%–55% of the biochar-specific removal target by 2050 (5 MtCO_2_e pa), equivalent to 0.6%–2.1% of the UK’s 2050 annual removal target (130 MtCO_2_e pa).^6^ From a technical perspective, it is crucial to develop more efficient production and supply strategies based on the spatial distribution characteristics of the feedstock. The southern and eastern regions of GB can implement biochar-based GGR more efficiently and cost-effectively. Additionally, biochar production can yield significant low-carbon energy byproducts.

Scaling this potential into deployable projects is likely to require targeted subsidies alongside market mechanisms. Public support can take the form of capital grants or investment tax credits to lower upfront costs, and performance-based payments for verified removals to reduce revenue risk. Support levels can be calibrated against a social carbon value, with the social cost of carbon providing an upper bound for public payments.

This study elucidates the value of straw-based biochar production in achieving net-zero targets, analyses the investment requirements for biochar, and highlights the technical and economic challenges of achieving GGR objectives through biochar production.

## 4 Discussion

This study employs a spatial optimization model to quantitatively analyze the marginal abatement cost and potential of straw-based biochar production in GB. The findings indicate that a national biochar framework could contribute 0.6–2.1% of the UK’s 2050 annual carbon removal target, with estimated biochar carbon sequestration costs ranging from £55 to £193 per t CO_2_e. Cost heterogeneity is driven by feedstock availability, transport distances and achievable plant scale, which vary across regions and between the low- and high-supply scenarios. Regions with denser feedstock availability, particularly in southern and eastern parts of GB, demonstrate higher biochar production efficiency due to shorter transport distances and economies of scale, making them optimal for cost-effective deployment. Placed in context, our estimates align with published values, including £135-£234 per t for UK straw-based biochar.^77^ Higher ranges reported for non-UK, nonstraw systems, for example £743-£933 per t for crop-residue biochar^78^ and £333-£1371 per t for orchard-waste biochar,^79^ reflect differences in feedstocks, geography and modeling choices. Prior UK estimates did not optimize supply chain geography, which likely contributed to broader and higher cost ranges. The spatial optimization model applied here improves the supply chain by concentrating throughput at fewer sites and reducing transport intensity, thereby lowering biochar cost.

From an economic perspective, feedstock supply constitutes approximately 70% of total production costs, making it the dominant cost driver in biochar production. Increasing the proportion of straw available for biochar from 22% (representing the share sold for market) to 57% (including all nonagricultural uses) substantially reduces the national average marginal cost from £74 to £64 per t CO_2_e. However, straw prices in the UK are subject to considerable fluctuation, with five-year market variability introducing uncertainty in marginal costs that can range from – 62%/+147% to – 24%/+55% in the low supply scenario, and from – 71%/+166% to – 24%/+54% in the high supply scenario. Expanding biochar production to utilize up to 57% of UK straw could place additional pressure on the market, particularly in regions where straw is already heavily used. This increased demand may drive up straw prices and intensify competition with sectors that purchase straw, including agricultural uses such as bedding and feed, even though on-farm retained straw is excluded from our supply scenarios. Consequently, the long-term feasibility and competitiveness of straw-based biochar will depend not only on technical factors, but also on how these market dynamics evolve over time.

Sensitivity analysis results reveal that marginal costs are highly sensitive to pyrolysis performance, particularly biochar yield and the HHV of bio-oil. A 20% reduction in either parameter results in an estimated 30% increase in cost, underscoring the importance of technological efficiency in cost control. In contrast, the external factors including transportation, labor, and infrastructure costs have relatively limited influence, with a 20% reduction in these parameters yielding only a 5% cost decrease.

From a policy standpoint, promoting straw-based biochar production can deliver meaningful carbon removal and avoided emission benefits at competitive costs. Across both supply scenarios, approximately 80% of the total carbon sequestration potential for straw biochar can be achieved at relatively low costs, making biochar competitive with some nature-based GGR approaches, such as habitat restoration. Realizing the full sequestration potential incurs higher costs but remains comparable to other engineered GGR technologies (BECCS, DACCS and enhanced weathering), underscoring biochar’s viability as both a cost-effective and scalable solution. To accelerate straw-based biochar deployment, targeted public support may include capital grants to reduce upfront costs and per-ton remuneration for verified removals. Support levels should be calibrated to the adopted carbon value.

This analysis focuses on supply side GGR costs and potentials. It does not evaluate where biochar would be most agronomically valuable to apply. Future work should integrate spatial agronomic suitability and application constraints with the supply chain to assess how demand centers align with feedstock supply and processing-site locations. This study redirects only sold straw to biochar production. In the low supply scenario this corresponds to sold for nonagricultural uses. In GB the role of domestic straw in power generation has diminished, with renewable output now met largely by imported wood pellets,^80,81^ so diverting these flows is not expected to materially affect electricity supply. In addition, bio-oil as the byproduct provides energy that partially compensates for foregone uses. In the high-supply scenario some sold straw may otherwise support agricultural applications after sale. Potential soil-organic-carbon effects from displacing those flows are not modeled and are acknowledged as a limitation.

Our results demonstrate that straw-based biochar production presents a viable method in the UK’s climate change mitigation pathway. Strategic optimization of feedstock allocation and processing site locations is crucial to maximize efficiency and minimize cost. Addressing these technical and economic challenges can enable straw-based biochar to play a pivotal role in achieving the net zero target.
